# Cost-Effectiveness of Bariatric Surgery versus Medication Therapy for Obese Patients with Type 2 Diabetes in China: A Markov Analysis

**DOI:** 10.1155/2019/1341963

**Published:** 2019-12-19

**Authors:** Bin Wan, Nan Fang, Wei Guan, Haixia Ding, Ying Wang, Xin Ge, Hui Liang, Xin Li, Yiyang Zhan

**Affiliations:** ^1^Department of Health Insurance Management, The First Affiliated Hospital with Nanjing Medical University, Nanjing, Jiangsu, China; ^2^Department of Health Policy, School of Health Policy and Management, Nanjing Medical University, Nanjing, Jiangsu, China; ^3^Department of General Surgery, The First Affiliated Hospital with Nanjing Medical University, Nanjing, Jiangsu, China; ^4^Department of Clinical Pharmacy, School of Pharmacy, Nanjing Medical University, Nanjing, Jiangsu, China; ^5^Center for Global Health, School of Public Health, Nanjing Medical University, Nanjing, Jiangsu, China; ^6^The First Affiliated Hospital with Nanjing Medical University, Nanjing, Jiangsu, China

## Abstract

**Aims/Introduction:**

The present study estimated the cost-effectiveness of bariatric surgery versus medication therapy for the management of recently diagnosed type 2 diabetes mellitus (T2DM) in obese patients from a Chinese health insurance payer perspective.

**Materials and Methods:**

A Markov model was established to compare the 40-year time costs and quality-adjusted life-years (QALYs) between bariatric surgery and medication therapy. The health-care costs in the bariatric surgery group, proportion of patients in each group with remission of diabetes, and state transition probabilities were calculated based on observed resource utilization from the hospital information system (HIS). The corresponding costs in the medication therapy group were derived from the medical insurance database. QALYs were estimated from previous literature. Costs and outcomes were discounted 5% annually.

**Results:**

In the base case analysis, bariatric surgery was more effective and less costly than medication therapy. Over a 40-year time horizon, the mean discounted costs were 86,366.55 RMB per surgical therapy patient and 113,235.94 CNY per medication therapy patient. The surgical and medication therapy patients lived 13.46 and 10.95 discounted QALYs, respectively. Bariatric surgery was associated with a mean health-care savings of 26,869.39 CNY and 2.51 additional QALYs per patient compared to medication therapy. Uncertainty around the parameter values was tested comprehensively in sensitivity analyses, and the results were robust.

**Conclusions:**

Bariatric surgery is a dominant intervention over a 40-year time horizon, which leads to significant cost savings to the health insurance payer and increases in health benefits for the management of recently diagnosed T2DM in obese patients in China.

## 1. Introduction

Diabetes is one of the major leading causes of mortality and disease burden. Mortality from diabetes doubled from 1990 to 2010 and increased to 1.3 million deaths worldwide in 2010 [1]. Diabetes is also a risk factor for vascular damage, including microvascular and macrovascular [2]. According to the latest report in the *International Diabetes Federation* (*IDF*) *Diabetes Atlas*, the prevalence of diabetes in adults is 9.1%, which implies that 415 million adults suffer from diabetes globally [3]. China has the highest number of people with diabetes, according to the latest published nationwide survey [4]. The prevalence of diabetes was 10.9%, and the estimated prevalence of prediabetes was 35.7% in 2013 [5]. The rapidly increasing prevalence of diabetes in China contributed to this worldwide diabetes pandemic.

In addition to the threat to health, diabetes also drives substantial direct health-care spending. The IDF estimated that 13% of total health-care expenditure in China, approximately US$25 billion in 2010, was attributable to diabetes [6]. A diabetes epidemic would further burden an already overloaded health insurance fund in China. The health-care costs for diabetes become a huge financial burden to patients, their families, and society as a whole.

Type 2 diabetes mellitus (T2DM) accounted for 93.70% of all types of diabetes in China [7], and its causes are not fully explained [2]. According to related studies, T2DM and obesity are linked strongly [8]. Weight control is the most important component of T2DM management [9]. However, clinical evidence indicates that current conventional therapies, including insulin, diet, exercise, behaviour modification, and oral agents, frequently fail to result in sustained relief for patients with morbid obesity [10], and increasing evidence reveals that the obese patients with T2DM benefit substantially from bariatric surgery [2, 10, 11]. For instance, one randomized, controlled trial by Schauer et al. [12], which included 150 patients who had T2DM, showed that the patients who underwent bariatric surgery had a greater mean percentage reduction from baseline in the glycated haemoglobin level than the patients who received medication therapy alone (2.1% vs. 0.3%, *P* = 0.003). A sustained reduction in the use of diabetes medications was also observed in Schauer et al.'s study, which demonstrated that the effects of bariatric surgery on glycaemic control were durable among patients with BMI of 27 to 34. Convincing long-term data show that weight loss decreased glycaemia effectively, which was captured in the follow-up of T2DM patients who received bariatric surgery [13]. Diabetes is virtually eliminated in this setting, with an average sustained weight loss of >20 kg [10, 13–16]. Available evidence indicates that bariatric surgery is cost-effective therapy for obese T2DM patients, and it is cost saving or dominant in some analyses [17, 18]. The National Institute for Health and Clinical Excellence (NICE) and IDF advocated for more widespread use of bariatric surgery in the management of obese patients with poorly controlled T2DM.

However, because bariatric surgery was originally developed to treat obesity, which is too often misconstrued as a “plastic” surgery, the government is not prepared to recommend reimbursement in China. The Chinese health-care system extended insurance coverage to nearly every citizen [6], but the reimbursement policy limits the use of bariatric surgery. One key concern has been described as uncertainty regarding the cost-effectiveness of bariatric surgery.

In recent years, bariatric surgery has been used to treat T2DM in obese patients in mainland China. One study in a tertiary hospital compared the remission of T2DM following treatment with laparoscopic sleeve gastrectomy (LSG) or laparoscopic Roux-en-Y gastric bypass (LRYGB) and compared the cost-effectiveness of LSG and LRYGB in T2DM patients [19]. However, no economic comparison was performed between bariatric surgery and conventional medication therapy because of the short period of bariatric surgery. Therefore, whether bariatric surgery is more cost-effective than lifelong medication for T2DM treatment for obese patients is not known.

The present study estimated the cost-effectiveness of bariatric surgery and conventional medication therapy as treatment for recently diagnosed T2DM in obese patients. We proposed a cost-effectiveness analysis to evaluate the economic values of the bariatric surgery based on clinical real-world data, which were extrapolated from the observed costs and outcomes over the 2-year follow-up to 40-year time horizon.

## 2. Methods

A 2-group retrospective cohort study was performed at The First Affiliated Hospital with Nanjing Medical University, which is a tertiary hospital in the city of Nanjing. The costs and outcomes of bariatric surgery and conventional medication therapy were calculated and presented as a cost-effectiveness ratio. The total costs include the 40-year time costs of direct health expenditures to treat T2DM. The results of effectiveness are expressed as quality-adjusted life-years (QALYs). Costs and QALYs were discounted at an annual rate of 5%. Costs are reported in 2015 China Yuan (CNY). The midyear 2015 currency exchange rate was 1 CNY to 0.16 USD. The analysis was performed from a third-party payer perspective over a forty-year horizon. Costs and health outcomes beyond the first year were discounted at an annual rate of 5%, which is consistent with Chinese guidelines for pharmacoeconomic evaluations, previous related studies, and China's consumer price index [19–22].

### 2.1. Markov Model Structure

A state transition Markov model using Microsoft Excel 2013 (Microsoft Inc., WA, USA) was constructed to assess the cost-effectiveness of bariatric surgery versus conventional medication therapy. Three health states were defined: T2DM remission state, T2DM state, and death state [11]. After taking drug treatment for one year, patients with the best control of fasting plasma glucose (FPG) and glycated haemoglobin (FPG ≤ 5.4 and glycated haemoglobin ≤ 6.0%) are in a state of T2DM remission, and those who did not meet the above criteria are in a state of T2DM [23].

**Figure 1:**
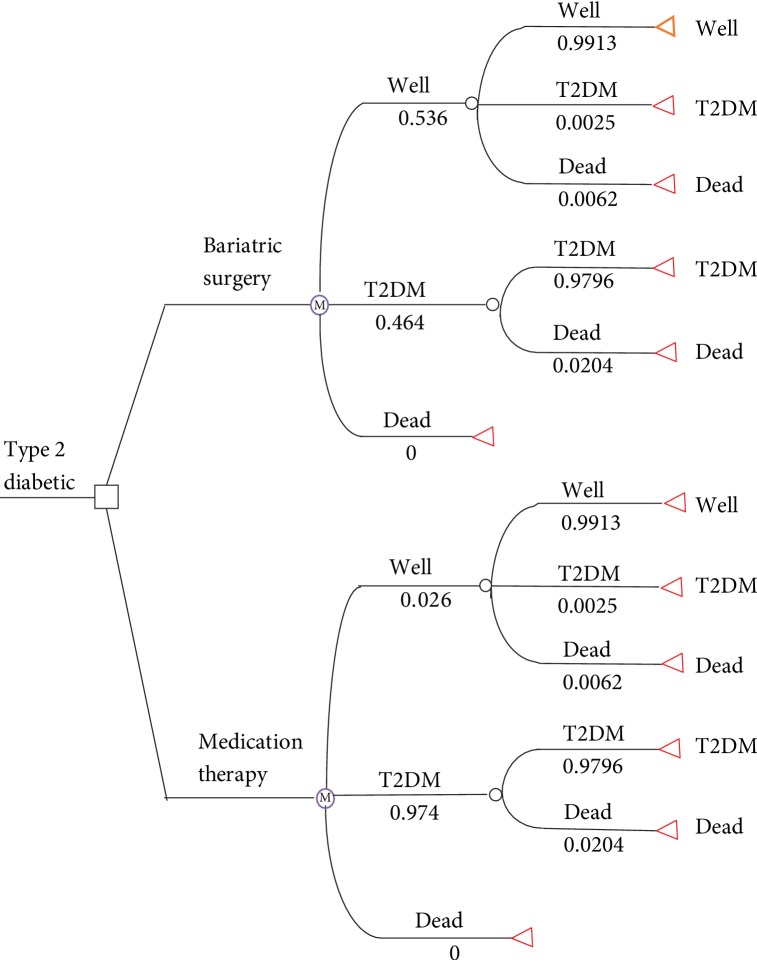
Markov model: health states and first cycle annual transition probabilities.

The model was applied from the end of a previous retrospective study. After morbidly obese patients underwent surgery or continued conventional medication therapy, they could develop T2DM, have T2DM remission, or die (Figure 1). The cycle length of the Markov model was one year. As time passed, patients could move between health states or stay in the previous state. Considering the average age of the base case patients and the average life expectancy in China, the Markov model was set to assess the 40-year cost-effectiveness of bariatric surgery versus conventional medication therapy.

### 2.2. Study Design and Patients

This study is a retrospective study, and the data of patients were collected from the clinical data repository (CDR) in the hospital and Nanjing Medical Insurance Database. We recruited patients suffering from T2DM with LRYGB surgery as the surgical group, and a propensity score matching (PSM) was used to identify the medication group of T2DM patients with conventional medication treatment. Patients were admitted between January 1, 2013, and December 31, 2015. LRYGB was performed in this study. LRYGB involved the creation of a small-volume gastric pouch which is anastomosed to the distal part of the alimentary limb. The gastric pouch of 20 ml was created. The lengths of biliopancreatic and roux limb were both 100 cm, and the size of gastrojejunal anastomosis was 1.5 cm. Mesenteric and Petersen defects were closed with a nonabsorbable suture. LRYGB could balance the safety and efficacy of surgical procedures, which was regarded as the “gold standard” in the surgery treatment of obesity and T2DM [24]. According to the guidelines in China, conventional medications of T2DM patients are basically metformin, sulfonylurea, and insulin [19]. The newer diabetes medications (e.g., GLP-1 agonists) were not included in the conventional medication therapy group.

The following inclusion criteria were used: patients aged between 18 and 65 years, recently diagnosed with type 2 diabetes (within 2 years) [25], with BMI ≥ 28 kg/m^2^, with fasting serum c-peptide in the lower 1/2 of the lower limits of normal [26, 27], and willing and able to comply with the research procedures set out in the program. The following criteria of exclusion were used: history of alcohol or substance abuse within 2 years, severe psychological disorder or mental illness, and poor medication compliance.

If a randomized controlled trial is not feasible, PSM can be used to create a randomization analog and minimize selection bias, controlling both known and unknown confounding variables and balancing population characteristics in observational studies [28, 29]. To balance the different characteristics of patients between the bariatric surgery and conventional medication groups, PSM was used to eliminate selection bias and match patients with multiple characteristics in observational groups. We simulated the random assignment of bariatric surgery and medication therapy groups by matching patients with medications to surgery patients who were similarly likely in the same group. First, we estimated the conditional probabilities that patients in the sample, given their BMI, gender, FPG, glycated haemoglobin, and other observed variables, thought to influence propensities. A logistic regression model was employed in the present study to generate the propensity score, which indicated a particular distance that measured similar observed characteristics between two groups of patients. The score is a predicted probability of patients who receive surgery based on their observed characteristics. All of the covariates were pretreatment patient characteristics. Second, we employed nearest-neighbour matching estimators to match patients with medications to similar bariatric surgery patients based exclusively on the values of their propensity scores. Calliper matching with a calliper value of 0.03 [30] and 1 : 1 matched pair was utilized in this study. If patients could not be matched to a counterpart, then they were discarded from the study.

### 2.3. Clinical Outcomes

The main clinical outcomes were extracted from the retrospective study and used in the first year of the mathematical modelling simulation based on data from a study on the trend of diabetes incidence and mortality in China, after which patients in different health states followed a natural course of risk factor progression [31].

### 2.4. Cost Data

Direct health expenditures are directly related to the medical intervention, which usually includes the prescription drugs, inpatient, outpatient, routine follow-up, and laboratory tests for each diabetic patient. As suggested in pharmacoeconomic guidelines [32–35], most economic evaluations do take into account direct health expenditures for treatment that are related to the medical intervention under evaluation, while ignoring other health expenditures altogether. Due to a third-party payer perspective (e.g., health insurer), direct health expenditures are most important in this study [32–35]. Therefore, only direct health expenditures for treatment of T2DM were included in the analysis. Direct nonmedical cost and indirect cost (e.g., travel and productivity costs to patients or caregivers) were not considered. Intervention and health-care costs for obese patients with T2DM in the bariatric surgery group were calculated based on observed resource utilization in real medical practices from the hospital information system, which included the cost of bariatric surgery procedures, hospitalization costs, and outpatient consultations. The corresponding costs in the conventional medication therapy group were derived from the urban employee basic medical insurance database of Nanjing, which included the costs of outpatient medical consultations and prescription medication.

### 2.5. Utility Data

Due to insufficient data on quality-adjusted survival associated with remission from T2DM, the QALYs of T2DM patients in the remission state were assumed the same as the general population. The mean utility equivalent to 0.95, which reflects the T2DM remission state, was sourced from Xie's study according to the EQ-5D scale [36]. The mean utility of patients with type 2 diabetes was 0.77, which was derived from an investigation of the quality of life of diabetic patients by Wang et al. in Jiangsu Province [37].

### 2.6. Transition Probabilities

The incidence of T2DM for relapse was applied to patients in the diabetes remission health state. The prevalence and incidence of T2DM were sourced from a 2017 global disease burden (GBD) study [38]. Due to a lack of sufficient evidence, it was assumed that the mortality risk for patients in remission from T2DM was the same as a healthy person. The transition probability for patients who moved from the remission state of T2DM to the death state was assumed to be all-cause mortality in nondiabetic populations.

We obtained annual mortality probabilities of patients with and without diabetes based on Magliano et al.'s study [39]. Magliano et al. proposed a formula for calculating the all-cause mortality rate for patients without diabetes (*μ*ND): *μ* = [*μ*ND × (1 − *p*)] + [*μ*ND × RR × *p*], where *μ* is the all-cause mortality rate for the total population, RR is the relative ratio of the population with diabetes relative to the population without diabetes, and *p* is the prevalence of diabetes. This formula can be revised as follows:

(1) $$\begin{array}{c} \mu \text{ND}=\frac{\mu }{1-p+p\text{RR}}. \end{array}$$

We proposed this formula for calculating the mortality rate for patients with diabetes (*μ*D) as follows: *μ*D = (*μb* − *μ*ND(*b* − *a*))/*a*, where *b* is the total national population and *a* is the population with diabetes. The result is

(2) $$\begin{array}{c} \mu \text{D}=\frac{\mu b-\left(\mu /\left(1-p+p\text{RR}\right)\right)\left(b-a\right)}{a}=\frac{\mu \left(b-\left(b-a\right)/\left(1+p\left(\text{RR}-1\right)\right)\right)}{a}. \end{array}$$

The all-cause mortality rate of the population with diabetes can be calculated based on the all-cause mortality rate of the population without diabetes, the prevalence of diabetes, the total population of China, and all-cause mortality of the total population. The all-cause mortality rate for the total population and the total national population in 2017 are derived from the National Bureau of Statistics of China. The RR was obtained from a study by Liu et al. on the trend of diabetes incidence and mortality in China [31]. Because the average age of patients in the base case is 43 years old, we used the mean of the RRs of patients 40-84 years old, and the weighted average was taken according to the gender ratio of patients in the base case. The model input data for medical cost and utility values and data sources are summarized in Table 1.

### 2.7. Sensitivity Analysis

To evaluate the robustness of the model results quantitatively, one-way sensitivity and probabilistic sensitivity analyses (PSA) were performed around critical factors that affected the cost-effectiveness of surgery and medication therapy. The key parameters in the model, such as costs, utilities, and discount rates, were changed within ±20% of the baseline value. A second-order Monte Carlo simulation (1,000 iterations) was conducted in PSA by inputting gamma distributions for the cost parameter and beta distributions for transition probability and health utility parameters. The willingness to pay (WTP) threshold was set at 3 times the GDP per capita (¥193,932/QALY). The results of sensitivity analysis were represented as a tornado diagram, scatter plots of incremental cost-effectiveness ratio (ICER), and cost-effectiveness acceptable curves.

### 2.8. Ethical Considerations

The Nanjing Medical University Institutional Review Board approved the study protocol (NJMUIRB (2018) 008). Written informed consent was obtained from all participants prior to data collection.

## 3. Results

### 3.1. Base Case Analysis

In total, 215 T2DM patients were enrolled in the study during the observation period: 134 patients in the LRYGB surgery group and 81 patients in the conventional medication group. However, there were significant differences in characteristics of patients between the two groups (Table 2). PSM was possible for five variables from 82 patients. Following PSM, no differences in baseline variables were observed between the groups. As a result, 41 pairs of patients were identified in the sample. There were no statistically significant differences in baseline values between the surgical group and the medication group (Table 2).

On one hand, in the unmatched cohort, after two years of surgery, 63 (47.0%) patients in the gastric surgery group were in a T2DM state, 71 (53.0%) patients were in T2DM remission state, and the death toll was 0 (Table 2). After two years of treatment, four patients were in the T2DM remission state in the general drug treatment group, which accounted for 4.9% of all patients. Seventy-seven patients were in a T2DM state, which accounted for 95.1% of all patients. The number of deaths was 0.

On the other hand, in the PSM matched cohort, after two years of surgery, 19 (46.4%) patients in the gastric surgery group were in a T2DM state, 22 (53.6%) patients were in a T2DM remission state, and the death toll was 0 (Table 2). After two years of treatment, only one patient was in the T2DM remission state in the general drug treatment group, which accounted for 2.6% of all patients. Forty patients were in a T2DM state, which accounted for 97.4% of all patients. The number of deaths was 0.

The initial cost per capita for patients with T2DM in the gastric surgery group included the average hospitalization and outpatient costs in the first year, which was 46,404.41 CNY per patient per year. The cycle cost per capita for patients included the average outpatient cost in the second and third years, and the cycle cost was 2,766.4 CNY.

In contrast, the initial cost per capita for patients with T2DM in the conventional drug treatment group included the average outpatient cost for the first year, which was 12,581.46 CNY per patient per year. The cycle cost per capita for patients included the average outpatient cost in the second and third years, and the cycle cost was 7,674.24 CNY.

### 3.2. Cost-Effectiveness Analysis

To compare and optimize the two intervention schemes, bariatric surgery and traditional medication therapy, the cost-effectiveness analysis method was adopted in this study. After 40-year cycles, the surgery group was in a dominant position compared to the traditional medication therapy group. As shown in Table 3, for the newly diagnosed obese diabetic patients, LRYGB surgery led to 13.46 QALYs gained and a cost-effectiveness ratio of 6,416.53 CNY/QALY; traditional medication therapy led to 10.95 QALYs gained and a cost-effectiveness ratio of 10,341.18 CNY/QALY. Compared to traditional drug-treated patients, patients treated with LRYGB surgery received an additional 2.51 discounted QALYs. Postdiscount costs per patient in the surgery group and the medicine group were 76,627.41 CNY and 134,287.73 CNY, respectively. For obese patients with T2DM, bariatric surgery is a more economical and effective alternative to traditional medicine therapy. Overall, bariatric surgery saved 57,860.32 CNY and produced an increase of 2.51 QALYs per patient.

**Figure 2:**
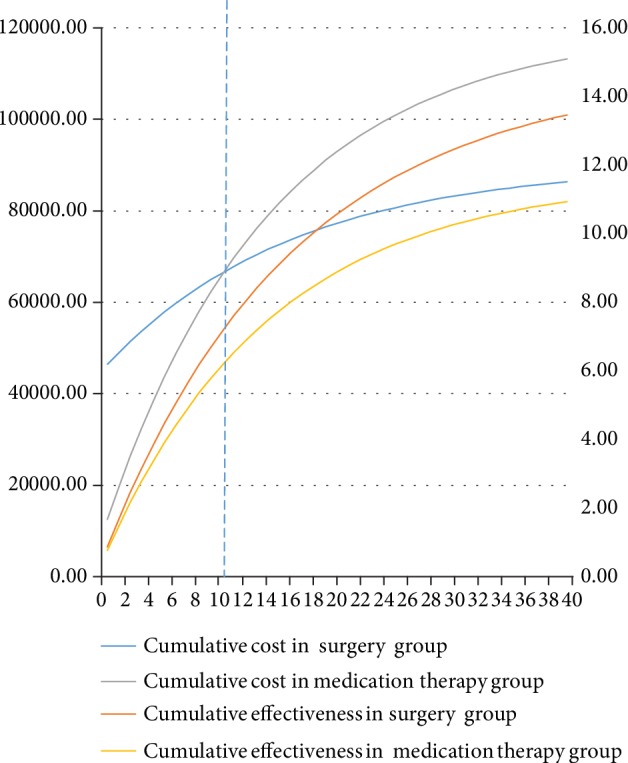
Trends in cost-effectiveness ratios between the bariatric surgery and conventional medication therapy group over 40 cycles. Cumulative eff. in the bariatric surgery therapy group was always higher than cumulative eff. in the conventional medication therapy group. In the middle of the cycle 10 and cycle 11, cumulative cost in the bariatric surgery therapy group and conventional medication therapy group began to cross.

The results of the 40-year time horizon are summarized in Figure 2. From cycle 1 to cycle 10, the bariatric surgery group incurred more costs and achieved more QALYs compared to the conventional medication therapy group. However, starting from cycle 11, bariatric surgery therapy began to be dominant. As shown in Figure 2, the cost of the bariatric surgery group was 67,837.36 CNY in cycle 11, and the utility was 7.60 QALYs; the cost of the conventional medication therapy group was 69,715.44 CNY in cycle 11, and the utility was 6.54 QALYs. The bariatric surgery therapy group had lower cost and higher utility compared to the conventional medication therapy group. Surgical intervention was a more economical choice, and bariatric surgery therapy was more cost-effective with the increasing cycle number. Therefore, conventional medication therapy was cheaper in the short term, but bariatric surgery therapy exhibited more economical efficiency in the long term.

### 3.3. Sensitivity Analyses

**Figure 3:**
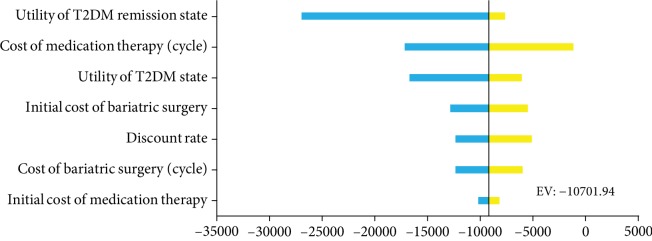
Tornado diagram of incremental cost-effective ratio for lower and upper bounds of input values. The range of ICER after varying input parameters.

**Figure 4:**
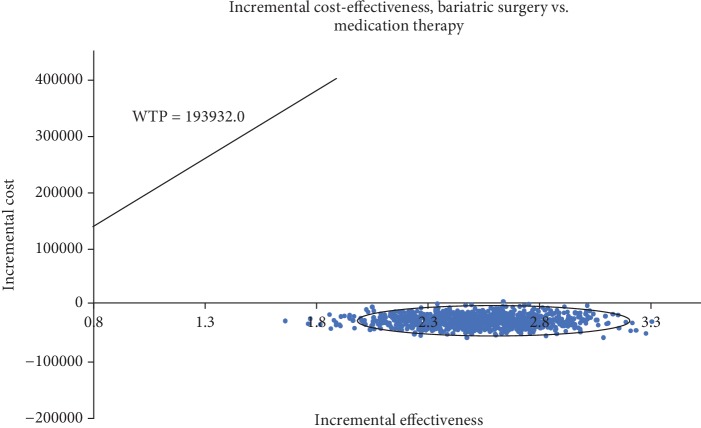
Probabilistic sensitivity analyses of bariatric surgery and conventional medication therapy.

**Figure 5:**
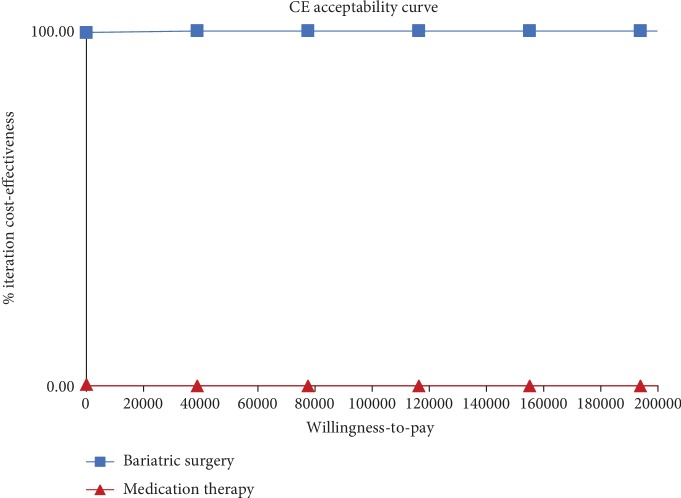
Cost-effectiveness acceptability curves.

Figure 3 shows the effects of varying each parameter on the cost-effectiveness ratio in tornado analyses. The utility of the T2DM remission state was a key factor that exerted the highest influence on the results, followed by the cost of medication therapy (cycle), utility of T2DM state, initial cost of bariatric surgery, discount rate, cost of bariatric surgery (cycle), and initial cost of medication therapy. Bariatric surgery therapy remained the predominant strategy regardless of changes in variables. The scattered points in Figure 4 show that the ICER of 1,000 simulations was lower than the threshold of WTP. Regardless of the WTP values changed, the probability of bariatric surgery being most cost-effective was 100% (Figure 5).

## 4. Discussion

To our knowledge, this study was the first report to evaluate the economy of bariatric surgery versus conventional medication therapy in China based on real-world data. From an economic perspective, the findings of this study show that bariatric surgery was superior for the management of diagnosed T2DM in obese patients due to cost savings and health benefits. These results are consistent with the overall estimates from other analyses [11, 17, 40–42]. These studies demonstrated the cost saving and high cost-effectiveness of surgery, which places bariatric surgery in a preferable position when health-care priorities must be established. A similar study from Australia by Keating et al. built on a within-trial cost outcome analysis and used a simple Markov model to compare the lifetime costs and QALYs between a surgery group and a medication group. The findings of this work demonstrated that surgical therapy was dominant to conventional medication therapy, which presented incremental costs of 2,400 AUD and incremental 1.2 QALYs [11]. However, the proportion of patients in the surgery group with remission of diabetes at 2 years in our study was significantly lower than that Keating et al.'s study. The clinical data in Keating et al.'s study was sourced exclusively from Dixon et al.'s study, which defined remission of diabetes as a fasting glucose level below 126 mg/dl and glycosylated haemoglobin (HbA1c) value below 6.2% without glycaemic medication [15]; the remission of diabetes was defined in our study as a fasting glucose level below 97.2 mg/dl and HbA1c value below 6.0% without medication. Considering inevitable cultural differences between different country contexts, the results of this study are broadly supportive of Keating et al.'s study. In China, a previous report by Tang et al. revealed that the evaluation of cost-effectiveness for each type of bariatric surgery performed via a Markov model yielded costs and QALYs. The cost-effectiveness ratio of gastric bypass surgery in Tang et al.'s study was 1,197.44 dollars per QALY [19], which is similar to the present study.

The use of a retrospective cohort in the present study provided information on the cost and treatment effects from the clinical real world, and an extensively validated economic model was used to predict long-term cost and health outcomes. The data sources of the cost and effectiveness in the present study differ from previous economic evaluation studies. All of the patients' data were collected from electronic databases such as Health Information System (HIS) and medical insurance database, which contributed to realize the traceability of data, minimize man-made mistakes, and reduce information bias in the process of data collection. As a retrospective study, the data were directly collected from clinical real world. We adopted a more comprehensive method to estimate health state transition by using the Markov model. Because of the significantly different economic frameworks and the specific model used, the outcomes from different studies are difficult to compare directly with the finding of the present study [17]. However, the similarity in ICERs from related studies increases the credibility of the health economic standpoint supporting bariatric surgery in obese T2DM patients.

## 5. Conclusions

The results from this real-world economic analysis showed that bariatric surgery is a dominant intervention over a 40-year time horizon compared to conventional medication therapy that can lead to significant health-care cost savings for health insurance payers and health gains in recently diagnosed T2DM in obese patients in China. From a cost perspective (disregarding the health gains and life expectancy benefits of diabetes remission), the results from the analytic model show that the surgical group is dominant over the medication group after 10 years, and the return on the utilization of bariatric surgery is recovered by the savings in costs and augmentations in QALYs. Therefore, the initial high costs of performing gastric bypass surgery should not be an obstacle to its reimbursement in obese populations with T2DM. On the other hand, the more widespread use of bariatric surgery in the management of obese patients with poorly controlled T2DM will benefit the Chinese health-care system because of the high reimbursement rates (approximately 85%) for diabetes treatment and the high prevalence of T2DM in China.

The present study had some limitations that are worth discussing. First, due to the lower calliper value of 0.03 in PSM, about two-thirds of patients in the surgery group and half of patients in the medication group could not be matched. Thus, only 41 pairs of patients were included in the model, which led to not many case numbers in this study. Second, some studies showed that the 30-day mortality associated with bariatric surgery was estimated at 0.1–0.3% [43], and it was described as “low” [44]. Due to the smaller sample size, no deaths occurred after surgery in this case. Third, the study did not pay special attention to different effects of different medications on the outcomes in the conventional medication therapy group. However, the medical treatment of T2DM in China is basically drug administration according to the guideline [19], which means that the different uses of medications would not impact cost obviously. Fourth, due to lack of the Official Guide of Bariatric Surgery before November 2014, only patients with their BMI > 32 kg/m^2^ could undergo bariatric surgery based on the Asia-Pacific Guide of Bariatric Surgery [45]. In this study, most of patients were admitted between January 2013 and November 2014, which led to higher average BMI in the surgery group (36.47 kg/m^2^) before propensity score matching. Only a small minority of patients with their BMI > 28 kg/m^2^ and <32 kg/m^2^ were admitted between December 2014 and December 2015 based on the Chinese Official Guide of Bariatric Surgery [27]. Therefore, the reliability of its results needs to be confirmed in a future study with a larger sample size and more rigorous design. Finally, the analytic model underestimated the possible benefits of surgical therapy. The analysis only captured the remission of T2DM as a benefit. It did not include potential obesity-related diseases. The inclusion of these health states may further increase its cost-benefit. Subject to these limitations, our findings show that bariatric surgery was more cost-effective; although short-term medical costs will increase if more individuals receive gastric bypass surgery, the added costs appear to provide a good value in the long term.

## Figures and Tables

**Table 1:** All cost and health inputs for the Markov model.

		Intervention group		
	All value (range)	Bariatric surgery therapy group value (range)	Conventional medication therapy group value (range)	Source
Cost (¥)				
Initial cost		46,404.41 (37,123.28-55,684.92)	12,581.46 (10,065.17-15,097.75)	The costs of the bariatric surgery therapy group were taken from the follow-up data; the costs of the conventional medication therapy group were derived from the basic medical insurance database of Nanjing employees
Incremental cost		2,766.41 (2,213.13-3,319.69)	7,674.24 (6,139.39-9,209.09)	
Utility weight reflecting				
With type 2 diabetes	0.77 (0.62-0.92)			Refer to [24]
Type 2 diabetes remission	0.95 (0.76-1.00)			Refer to [25]
Transition probabilities				
Annual probability for relapse to type 2 diabetes	0.0025 (0.0020-0.0031)			Refer to [11], National Bureau of Statistics of China
Annual mortality probability: type 2 diabetes (age 42–82 years)	0.0204 (0.0163-0.0245)			Refer to [11, 19, 21, and 22]
Annual mortality probability: type 2 diabetes remission (age 42–82 years)	0.0062 (0.0050-0.0074)			Refer to [11, 19, 21, and 22]
Rate (%)				
Rate	5 (4-6)			

**Table 2:** Baseline values for patients in the bariatric surgery therapy group and the conventional medication therapy group.

	Unmatched cohort		Propensity score-matched cohort			
Demographic information	Bariatric surgery therapy group	Conventional medication therapy group	*P* value	Bariatric surgery therapy group	Conventional medication therapy group	*P* value
Demographic						
Sample size (patients)	134	81		41	41	
Sex (female)	65	27	0.029^†^	24	22	0.66^†^
Age (years)^a^	39.05 ± 12.03	42.54 ± 12.04	0.040^‡^	41.54 ± 11.85	42.90 ± 11.18	0.60^‡^
BMI (kg/m^2^)^a^	36.47 ± 8.15	33.65 ± 4.12	0.001^‡^	32.73 ± 4.52	34.34 ± 3.26	0.07^‡^
Clinical data						
Fasting plasma glucose^a^	9.74 ± 2.98	8.91 ± 2.31	0.021^‡^	8.90 ± 2.43	8.54 ± 2.47	0.52^‡^
Glycated haemoglobin^a^	8.49 ± 1.46	7.93 ± 1.84	0.033^‡^	9.12 ± 1.55	9.67 ± 1.70	0.13^‡^
Diabetes status (after 2 years)						
No. with T2DM remission (patients)	71	4	<0.0001^†^	22	1	<0.0001^†^
No. with T2DM (patients)	63	77		19	40	
No. of death (patients)	0	0		0	0	

^a^Values are presented as the mean ± standard deviations. ^†^*χ*^2^ test; ^‡^*t*-test.

**Table 3:** Model results.

	Intervention group		
	Bariatric surgery therapy group	Conventional medication therapy group	Difference
Undiscounted			
Total cost (¥)	132,958.29	221,118.48	-88,160.19
QALYs	28.20	21.86	6.34
Discounted at 5% for both costs and benefits			
Total cost (¥)	86,366.55	113,235.94	-26,869.39
QALYs	13.46	10.95	2.51
Cost-effectiveness			
Cost per QALY (discounted)	6,416.53 (dominant)	10,341.18	

## Data Availability

The clinical data used to support the findings of this study are restricted by the Nanjing Medical University Institutional Review Board in order to protect patient privacy. Data are available from the corresponding authors for researchers who meet the criteria for access to confidential data.
